# Diagnostic value of serum COMP and ADAMTS7 for intervertebral disc degeneration

**DOI:** 10.1186/s40001-024-01784-w

**Published:** 2024-03-25

**Authors:** Jing-Yu Ding, Xu Yan, Ren-Jie Zhang, Hua-Qing Zhang, Liang Kang, Chong-Yu Jia, Rick F. Thorne, Xiao-Ying Liu, Cai-Liang Shen

**Affiliations:** 1 Department of Orthopedics and Spine Surgery, The First Affiliated Hospital of Anhui Medical University, 218 Jixi Road, Hefei, 230022 Anhui China; 2 Laboratory of Spinal and Spinal Cord Injury Regeneration and Repair, The First Affiliated Hospital of Anhui Medical University, 218 Jixi Road, Hefei, 230022 Anhui China; 3 Anhui Province Research Center for the Clinical Application of Digital Medical Technology, The First Affiliated Hospital of Anhui Medical University, Hefei, 230022 Anhui China; 4 School of Life Sciences, Anhui Medical University, 81 Meishan Road, Hefei, 230032 Anhui China; 5 Henan International Joint Laboratory of Non-Coding RNA and Metabolism in Cancer, Henan Provincial Key Laboratory of Long Non-Coding RNA and Cancer Metabolism, Translational Research Institute of Henan Provincial People’s Hospital and People’s Hospital of Zhengzhou University, Zhengzhou, Henan China

**Keywords:** Intervertebral disc degeneration (IVDD), Cartilage oligomeric matrix protein (COMP), A disintegrin and metalloproteinases with thrombospondin motifs7 (ADAMTS7), Biomarkers, Early diagnosis

## Abstract

**Objective:**

Intervertebral disc degeneration (IVDD) is a major cause of morbidity and disability. Our study aimed to investigate the potential of cartilage oligomeric matrix protein (COMP) and ADAMTS7 (A disintegrin and metalloproteinases with thrombospondin motifs 7) as biomarkers for IVDD together with their functional relationship.

**Methods:**

IVD tissues and peripheral blood samples were collected from IVDD rabbit models over 1–4 weeks. Tissues and blood samples were also collected from clinical patients those were stratified into four equal groups according to Pfirrmann IVDD grading (I–V) with baseline data collected for each participant. COMP and ADAMTS7 expression were analyzed and biomarker characteristics were assessed using linear regression and receiver operating curve (ROC) analyses.

**Results:**

COMP and ADAMTS7 expression increased in tissues and serum during IVDD progression. Serum COMP (sCOMP) and serum ADAMTS7 (sADAMTS7) levels increased in a time-dependent manner following IVD damage in the rabbit model while significant positive correlations were detected between sCOMP and sADAMTS7 and Pfirrmann grade in human subjects. ROC analysis showed that combining sCOMP and sADAMTS7 assay results produced an improved diagnostic measure for IVDD compared to individual sCOMP or sADAMTS7 tests. In vitro assays conducted on human cell isolates revealed that COMP prevented extracellular matrix degradation and antagonized ADAMTS7 expression although this protective role was uncoupled under microenvironmental conditions mimicking IVDD.

**Conclusions:**

Increases in circulating COMP and ADAMTS7 correlate with IVDD progression and may play regulatory roles. Assays for sCOMP and/or sADAMTS7 levels can discriminate between healthy subjects and IVDD patients, warranting further clinical assessment.

**Supplementary Information:**

The online version contains supplementary material available at 10.1186/s40001-024-01784-w.

## Introduction

One of the main root causes of health expenditure and financial burden over the past decades involves low back pain (LBP) [1]. Among the predisposing factors leading to LBP, intervertebral disc degeneration (IVDD) represents its primary pathological cause [2]. Current clinical treatments for IVDD involve drug and surgical interventions to control symptoms and minimize disability, although these approaches often lead to various complications, with the additional incumbrance suggesting questionable efficacy [3]. The prospects of developing a biological means for repairing and regenerating degraded intervertebral discs (IVDs) are therefore highly attractive, with approaches including cell therapy [4, 5], biological factor regulation [6, 7], non-coding RNA therapy [8, 9] and gene therapy [10–12]. Moreover, such non-surgical interventions have the potential for disrupting the etiology of IVDD at an early stage, thus delaying or preventing further deterioration, and even reversing the damage. On this basis, the early diagnosis of IVDD is particularly critical for improving disease management.

At present, clinical diagnoses of IVDD are made through medical history and physical examination along with follow-up imaging examinations. Regarding the latter, magnetic resonance imaging (MRI) is considered the best non-invasive albeit expensive approach for definite diagnosis [13, 14]. Different IVD signals and heights shown on MRI images are used for clinical classification, although these measures do not accurately reflect the stage of IVDD [15]. Moreover, since histopathological changes always precede imaging changes, the accurate early diagnosis of IVDD is still challenging. Even with the advanced capabilities of 3.0 T MRI, histopathological changes in the IVD cannot always be detected [14, 16]. Therefore, it is essential to develop sensitive and specific early approaches that will supplement IVDD diagnoses. In this regard, specific biomarkers are necessary for diagnosis in a wide range of pathologies [17, 18]. Studies of IVDD showed that the degradation products of extracellular matrix (ECM) and the levels of some inflammatory mediators are increased in the circulation of patients [19, 20]. Importantly, these prospective biomarkers can be detected before the pathological changes in the IVD. These findings imply that molecular markers could outperform MRI in the early diagnosis of IVDD [21], although the critical question involves which biomarkers are best suited for transposition into clinical tests.

Similar to osteoarthritis (OA) and rheumatoid arthritis (RA), IVDD is a degenerative disease of the cartilage manifested as ECM degradation [22]. Cartilage homeostasis in the IVD maintained through ECM damage repair pathways with the essence of IVDD pathogenesis being an imbalance between anabolic and catabolic processes. This in turn changes the ECM composition of the IVD, gradually leading to cartilage degradation [23]. Among the key players in maintaining cartilage integrity is the cartilage oligomeric matrix protein (COMP), a pentameric protein linked by disulfide bonds belonging to the platelet cadherin family. COMP binds to the central ECM components in cartilage, including collagen I, collagen II, collagen IX, fibronectin, and proteoglycans, to stabilize the composition of the ECM [24–26]. Studies in OA, RA and reactive arthritis have shown that COMP is degraded and released into serum and/or urine in the early pathogenic stages, with serum COMP (sCOMP) levels distinguishing between OA and healthy individuals and reflecting disease severity [27–30]. Moreover, recent findings in the animal model of IVDD reported that sCOMP was upregulated during the progression of IVDD [31]. These instructive findings suggest that the relationship between COMP release and the clinicopathological characteristics of IVDD needs to be further explored.

Of further relevance to this report is the mechanism whereby COMP and other ECM components are degraded. Featuring prominently in these processes are members of the ADAMTS (A Disintegrin And Metalloproteinase with Thrombospondin motifs) metalloproteinase family. In specific, ADAMTS7 directly binds to and degrades COMP, therefore implicating its dysregulation in the pathology of degenerative diseases [32, 33]. Notably, ADAMTS7 is expressed in bone, joints and synovium and the development of IVDD is accompanied by the upregulation of ADAMTS7 [34]. Together this proposes that COMP degradation by ADAMTS7 contributes to IVDD pathogenesis. Furthermore, whether higher concentrations of soluble ADAMTS7 are detectable in circulation during IVDD progression remains a further question to address.

In this study, we investigated the relationship between the levels of tissue and circulating COMP and ADAMTS7 in IVDD progression in both animals and human LBP subjects. For the former, we employed annulus fibrosus puncture in rabbits to mimic disc injury, a widely used model system of IVDD [35, 36]. Findings from the animal model showed induction of COMP mRNA and protein expression following mechanical disruption of the IVD together with increases in serum COMP and ADAMTS7 levels. This phenomenon was reflected in a cohort of LBP patients that progressive pathological damage assessed by Pfirrmann grade was associated with higher COMP/ADAMTS7 levels in both IVD tissues and serum. Manipulating COMP levels in primary isolates of nucleus pulposus (NP) cells from human subjects confirmed that COMP acts to stabilize ECM components, partially through dampening ADAMTS7 expression. And although COMP was strongly induced during acidification of the culture, a state that mimics the IVD microenvironment during IVDD etiology, its protective role in ECM stability was negated. Last, analysis of clinicopathological measures including Pfirrmann grade, the Oswestry Disability Index (ODI) and visual analog scale (VAS) pain scores to evaluate the diagnostic potential of sCOMP and sADAMTS7 revealed that upregulation of sCOMP and sADAMTS7 may provide indications of disease presence and/or severity. Follow-on receiver operating characteristic (ROC) analysis indicated combining sCOMP and sADAMTS7 levels showed good discriminatory potential in the diagnosis of IVDD.

## Materials and methods

### Animal model of IVDD

Twenty-five New Zealand white rabbits weighing approximately 3.5 kg were employed to model IVD damage responses using the annulus fibrosus puncture (AFP) technique. Each rabbit underwent puncture of four exposed intervertebral discs (L2/3 to L5/6) using 18-G needles to a consistent depths of 5 mm. The needle was inserted at the disc's center through the annulus fibrosus (AF) into the NP and held for 5 s. Unperforated discs above and below the exposed discs served as control discs. Blood samples were collected from the auricular vein of nine animals before surgery and at 1-, 2- and 4-week timepoints after puncture. In additional, three rabbits were killed at 1-, 2- and 4-week timepoints for subsequent histological analyses, respectively (Additional file 1: Figure S1). All animal experiments were conducted with the approval of the Laboratory Animal Center of Anhui Medical University (No. 20170423), in accordance with the International Guiding Principles for Animal Research, under appropriate guidance and supervision.

### Patient sample collection

One hundred patients receiving surgical interventions for LBP were recruited to the study. Detailed baseline information was collected including clinical severity scores, VAS and ODI while Pfirrmann grading was applied to evaluate the degree of IVDD. Further screening was applied using the exclusion criteria: patients with OA, RA; patients with surgical intervention or intra-articular steroid injections in the past 6 weeks and analgesics in the past 2 weeks. This excluded 16 patients to derive a total of 84 patients, with 21 patients classified by Pfirrmann grading as I–II, III, IV and V grades, respectively. Presurgical peripheral blood samples were collected for ELISA along with NP tissues from surgery for histological examination and primary cell culture.

### Ethics statement involving human subjects

Samples were obtained from patients undergoing surgery at the First Affiliated Hospital of Anhui Medical University. The study adhered to the ethical standards set by the responsible committee on human experimentation (institutional and national) and followed the Helsinki Declaration of 1975, as revised in 2000. Written informed consent, accompanied by patient or guardian signatures, was obtained prior to tissue collection. This study was approved from the institutional review board of Anhui Medical University (No. 2017372).

### Histological evaluation

After fixation in 4% formaldehyde solution, rabbit lumbar spines were decalcified for 20 days. Human NP and rabbit spine tissues were subjected to paraffin embedding and 5-µm slices sections prepared for immunohistochemistry (IHC; see below) and histological staining. Human NP tissues were stained with Hematoxylin–Eosin (HE) and Alcian blue. Rabbit IVDs were stained with HE and Safranin O-Fast Green (SO). Histological scoring was implemented according to a previous study [8] to assess degenerative changes in 5 categories with scores ranging from 0 to 15 points.

### Cell culture and treatment

Human NP tissue was isolated from the surrounding fibrous tissue and washed with sterile phosphate-buffered saline (PBS, Sigma) before mincing into approximately 0.5 × 0.5 × 0.5 mm^3^ fragments using sterile ophthalmic scissors. The fragments were digested with a 2 mg/mL solution of type II collagenase (Sigma-Aldrich, USA) at 37℃ for 6 h, followed by centrifugation at 2000 rpm for 5 min. Cell pellets were resuspended and cultured in DMEM medium (HyClone, USA) for 24 h to remove non-adherent and non-viable cells. The isolates were passaged weekly using 0.25% trypsin–EDTA (Biosharp), with cells from the second passage (P2) utilized for the in vitro experiments. The culture medium was replaced with acid-conditioned medium prepared as previously described [5], or serum-free DMEM for 12 h before treatment with 0–6 μg/mL recombinant human COMP (rhCOMP; R&D Systems, Minneapolis, MN).

### Immunohistochemistry

Slides were deparaffinized with xylene and rehydrated using graded alcohols. Ethylenediaminetetraacetic acid (0.1 mol/L, pH 9.0) solution was used for antigen retrieval/repair before sequentially quenching with H_2_O_2_ solution, blocking using normal goat serum, and incubation with anti-COMP (1:500, Abcam, USA) or anti-ADAMTS7 (1:500, Proteintech, China) overnight at 4℃. The sections were then incubated with an HRP-conjugated secondary antibodies and immunocomplexes detected using a DAB horseradish peroxidase color development kit (Proteintech, Wuhan, China). After washing, the slides were counterstained with hematoxylin and whole slide images acquired with a Panoramic tissue cell quantitative analysis system (Tissue Gnostics GmbH, Austria).

### Immunofluorescence

Cultured NP cells underwent immunofluorescence staining with anti-COMP antibodies (1:200 dilution, Abcam, USA) or ADAMTS7 antibodies (1:200 dilution, Proteintech, China) overnight at 4℃. Subsequently, the cells were incubated with the species-matched fluor-labeled secondary antibodies (Invitrogen) for 1 h at room temperature before counterstaining with DAPI. Cells were examined using epifluorescence microscope (Leica, Germany).

### Enzyme-linked immunosorbent assay (ELISA)

Serum samples were subjected to ELISA assays to measure sCOMP and sADAMTS7 levels according to the manufacturers recommended protocols (MM-1181H1, MM-82616O1, MM-61678H1 and MM-82819O1, Jiangsu Meimian Industrial Co., Ltd). In brief, primary antibodies were used to coat microtiter plate wells before the addition of 1:5 diluted serum samples, followed by HRP-conjugates and TMB substrate solution. Reactions were terminated by sulfuric acid solution before measuring OD at 450 nm. The concentrations of sCOMP and sADAMTS7 were determined against a standard curve constructed using recombinant proteins.

### RNA extraction and RT-qPCR

Total RNA was extracted using E.Z.N.A. Total RNA Kit 1 (Omega BIO-TEK, USA), and PCR analysis was performed using the NovoStart® SYBR qPCR SuperMix Plus (Novoprotein, China). The 2^−ΔΔCT^ method was used to analyze the relative mRNA expression in comparison to the reference gene GAPDH. The primer sequences for amplifying COMP and ADAMTS7 and internal control GAPDH were as follows: COMP (F: 5′-CGTGCGGCCCCTGCTCCACTGCG -3′, R: ACCTGCTTGTTGGCCTTGGCGAAGCCA-3′); ADAMTS7 (F: 5′-TCAGTGCTCAGTGACATGTGGGGA-3, R: 5′-CGTTGAAGAGCTCGTGGCTGGA-3′); GAPDH (F: 5′-GGAGCGAGATCCCTCCAAAAT-3′, R: 5′-GGCTGTTGTCATACTTCTCATGC-3′).

### Cell transfection

Small interfering RNAs (siRNAs) specifically targeting COMP (sense: 5'-AGAAACUUGAGCUGUUGAUGCC-3') and a non-targeting negative control (NC) siRNA (GENERAL BIOL, Anhui, China) were used in knockdown experiments. In brief, NP cells were seeded in 6-well plates and transfected with 50 nM of siRNAs using Lipofectamine 2000 Reagent (Thermofisher Scientific, MA, USA) according to the manufacturer's instructions.

### Total protein extraction and Western blotting

Extraction of total protein and Western blotting analyses were performed as described previously [37]. Primary antibodies used included COMP (Abcam ab300555, 1:1000), Aggrecan (Abcam ab3778, 1:1000), Collagen II (Immunoway YT1022, 1:1000) and GAPDH (Proteintech 60,004–1-lg, 1:10,000).

### Imaging examination and analysis

One, two and four weeks after the AF acupuncture procedure, 9 rabbits were randomly selected for X-ray and MRI examination. Lumbar computed radiography imaging was performed by Digital X-ray Imaging System (Siemens, Berlin, German), and MRI tests were performed via a 3.0 T system (Siemens, Berlin, German). The changes in IVD height were evaluated by the disc height index (DHI) which was expressed as the mean of the 3 measurements from midline to the boundary of the central 50% of disc width divided by the mean of the 2 adjacent vertebral body heights. Changes in the DHI of punctured discs were expressed as a percentage (%DHI = post-punctured DHI/pre-punctured DHI × 100). All results were analyzed independently by two radiologists and the mean values used.

### Definition of clinical severity

The clinical severity of 84 patients was evaluated using ODI and VAS scores. Pain level was assessed on a 0–10 VAS, with 0 indicating no pain and 10 representing extremely unbearable pain. The ODI consisted of 10 items that measured the impact of symptoms on daily activities, including pain intensity and functionality during tasks such as traveling, sexual activity, sitting, walking, lifting, sleeping, standing, and personal hygiene. ODI scores were graded on scales ranging from 0 to 100.

### Statistical analysis

Continuous variables were presented as mean ± standard deviation (SD) or median, while categorical variables were expressed as number (percentage). Student’s *t* test was employed to compare the differences between two groups. One-way repeated measures ANOVA was conducted to compare the concentration of sCOMP and sADAMTS7 among the different patient groupings and Bonferroni post-hoc tests were used for pairwise comparisons. Multivariate linear regression analysis was performed to investigate the relationships between VAS, ODI, Pfirrmann grade (as a continuous variable) and sCOMP, sADAMTS7, adjusting for age, sex, BMI, and hypertension (Additional file 1: Figure S2). Stratified analyses were also conducted based on gender (male and female), BMI (normal and overweight/obesity), and hypertension (normal and abnormal) to examine the correlation between independent variables (VAS, ODI, Pfirrmann grade) and outcome variables (sCOMP and sADAMTS7). ROC analysis was performed for the evaluation of the diagnostic potential of COMP and ADAMTS7 for IVDD. The area under the curve (AUC) was calculated as the diagnostic test in which 1 indicates a perfect discrimination. Sensitivity and specificity according to the cut-off values of the biomarkers related to IVDD were calculated. Data analyses were performed using SPSS version 23.0 for Windows (SPSS Inc., Chicago, IL, USA), and 2-side *P* values < 0.05 were considered statistically significant.

## Results

### Characterization of the animal IVD degeneration model

**Fig. 1:**
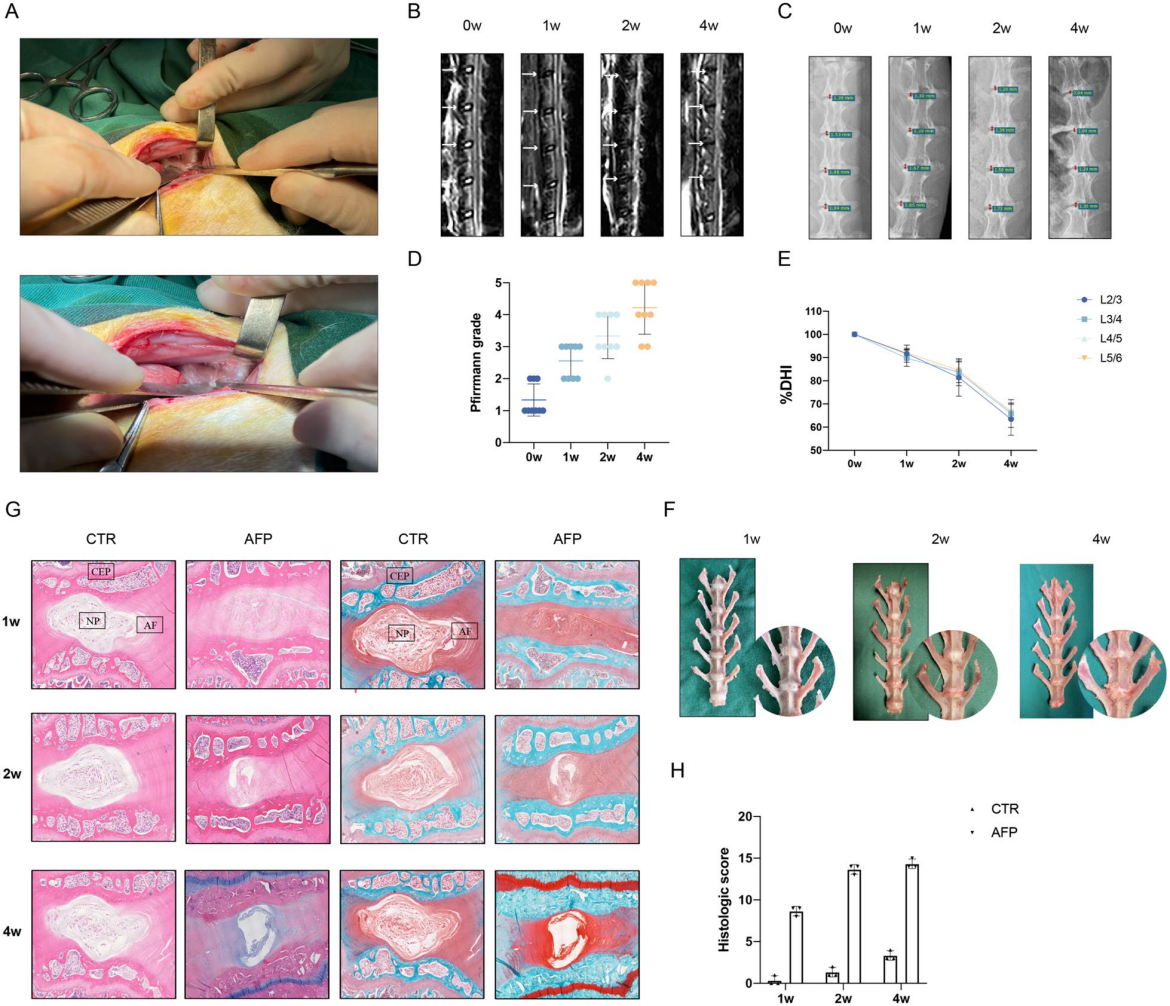
Construction of intervertebral disc degeneration model. **A** The rabbit model of intervertebral disc degeneration (IVDD) was completed by acupuncture. **B** The four representative MRI images including four model discs at the different time points and **D** quantitative classification based on the Pfirrmann grade, *n* = 9. **C** The representative X-ray film images and **E** %DHI fold line including four model discs at the different time points, *n* = 9. **F** Pathological changes of gross specimens after 1, 2, and 4 weeks of acupuncture. **G** H&E staining (Left) and SO staining (Right) showed severe IVDD in the AFP group. **H** Evaluation of IVDD by histological score based on SO staining.* n* = 3

After acupuncture of four consecutive lumbar IVDs (L2/3 to L5/6) in New Zealand white rabbit (Fig. 1A), the results of consecutive MRI scans over 0–4 weeks revealed progressive degeneration, with the NP structure gradually changing from a uniform, bright white to being disordered and blackened. The boundary of the NP and AF was gradually lost, with the signal intensity of the MRI T2 phase decreasing (Fig. 1B). Accompanying X-ray imaging documented corresponding changes in the height of the IVD in the punctured segments (Fig. 1C). Quantitative analysis of the image data indicated progressive increases in Pfirrmann scores (Fig. 1D) along with statistically significant decreases in DHI occurring 2 weeks after the procedure (Fig. 1E).

Furthermore, since pathological changes in IVDD always precede imaging differences, it was important to further characterize the model with respect to its histology. At the gross level, obvious osteophytic growth was evident after 4 weeks compared to spines dissected at 1 or 2 weeks post-insult (Fig. 1F). Further analysis of sections stained with H&E to examine disc structures or using the cartilage stain Safranin O-Fast Green confirmed that no pathological changes in the NP were evident in control groups across time points. In contrast, disks following acupuncture treatment showed varying degrees of degeneration demonstrated by dissolution of the boundary between the AF and NP, along with annular disruption and gradual time-dependent decreases in the numbers of NP cells (Fig. 1G). Together these data indicated that the model recapitulates the degenerative changes in imaging associated with human IVDD.

### Circulating and tissue levels of COMP and ADAMTS7 are increased during IVDD pathogenesis in rabbits

**Fig. 2:**
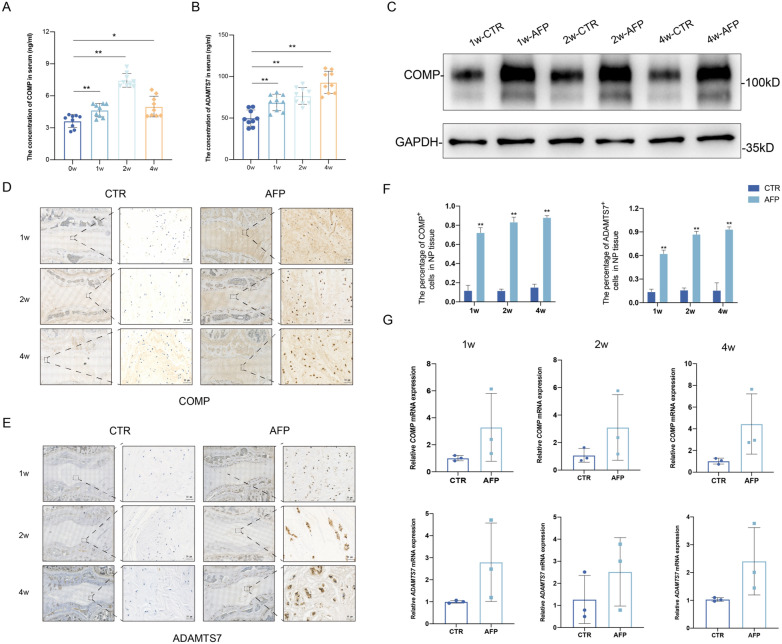
Circulating and tissue levels of COMP and ADAMTS7 among different time points during rabbit models. ELISA assays measuring. **A** sCOMP and **B** sADAMTS7 at 0 week (before surgery), 1 week, 2 weeks and 4 weeks after acupuncture. One-way repeated measures ANOVA was conducted to compare the concentration of sCOMP and sADAMTS7 among the different patient groupings, and Bonferroni post-hoc tests were used to pairwise comparisons, ^*^ indicating *P* ≤ 0.05 compared to 0w group; ^**^ indicating *P* ≤ 0.01 compared to 0w group. **C** Western blotting analyses of the expression of COMP in rabbit IVD tissues in different time points. IHC stainings of COMP (**D**) and ADAMTS7 (**E**) in rabbit IVD tissues. **F** The expression level of COMP and ADAMTS7 were quantified as positive rate, results were shown as mean ± SD. *P* value was calculated with *t* test. ^**^ indicating *P* ≤ 0.01 compared to CTR. **G** RT-qPCR analysis of COMP and ADAMTS7 expression in rabbit IVD tissues at different time points. Scale bar, 50 µm

To investigate the premise that soluble forms of COMP and ADAMTS7 are prospective IVDD biomarkers, we detected their levels in serum after collecting peripheral blood from the rabbit IVDD model. Using ELISA assays, we observed that the sCOMP levels progressively increased at 1 and 2 weeks after treatment, while showing a decline at 4 weeks although still higher than before surgery (Fig. 2A). Similarly, sADAMTS7 levels also increased over time although without any decrease 4 weeks post-operation (Fig. 2B).

To assess if the altered levels of circulating COMP and ADAMTS7 were reflected in the changes within damaged discs, we compared their expression in NP tissues. Indeed, using Western blotting we found a striking increase in COMP levels 1 week after surgery that was further increased over time (Fig. 2C). Moreover, direct observation using immunohistochemistry showed that COMP was mainly distributed in the ECM of the nucleus pulposus cells, with higher expression in degenerative compared to normal segments (Fig. 2D). Staining for ADAMTS7 increased in an analogous manner, with reactivity mainly evident in the cytoplasm, with notably higher expression in acupunctured segments relative to the controls (Fig. 2E). Using qPCR to measure mRNA levels in NP tissues, we found a general trend showing COMP and ADAMTS7 transcript levels was increased in the diseased condition (Fig. 2G), although less obvious than protein changes.

Collectively these results support the notion that progressive upregulation of sCOMP and sADAMTS7 in IVDD may provide indications of disease presence and/or severity. Furthermore, the positive associations between COMP/ADAMTS7 expression changes in NP tissues and the levels of circulating sCOMP/sADAMTS7 suggest the basis for their release into circulation. It was next important to examine evidence for the proposed mechanism in humans.

### Circulating and tissue levels of COMP and ADAMTS7 are increased during IVDD pathogenesis in humans

**Fig. 3:**
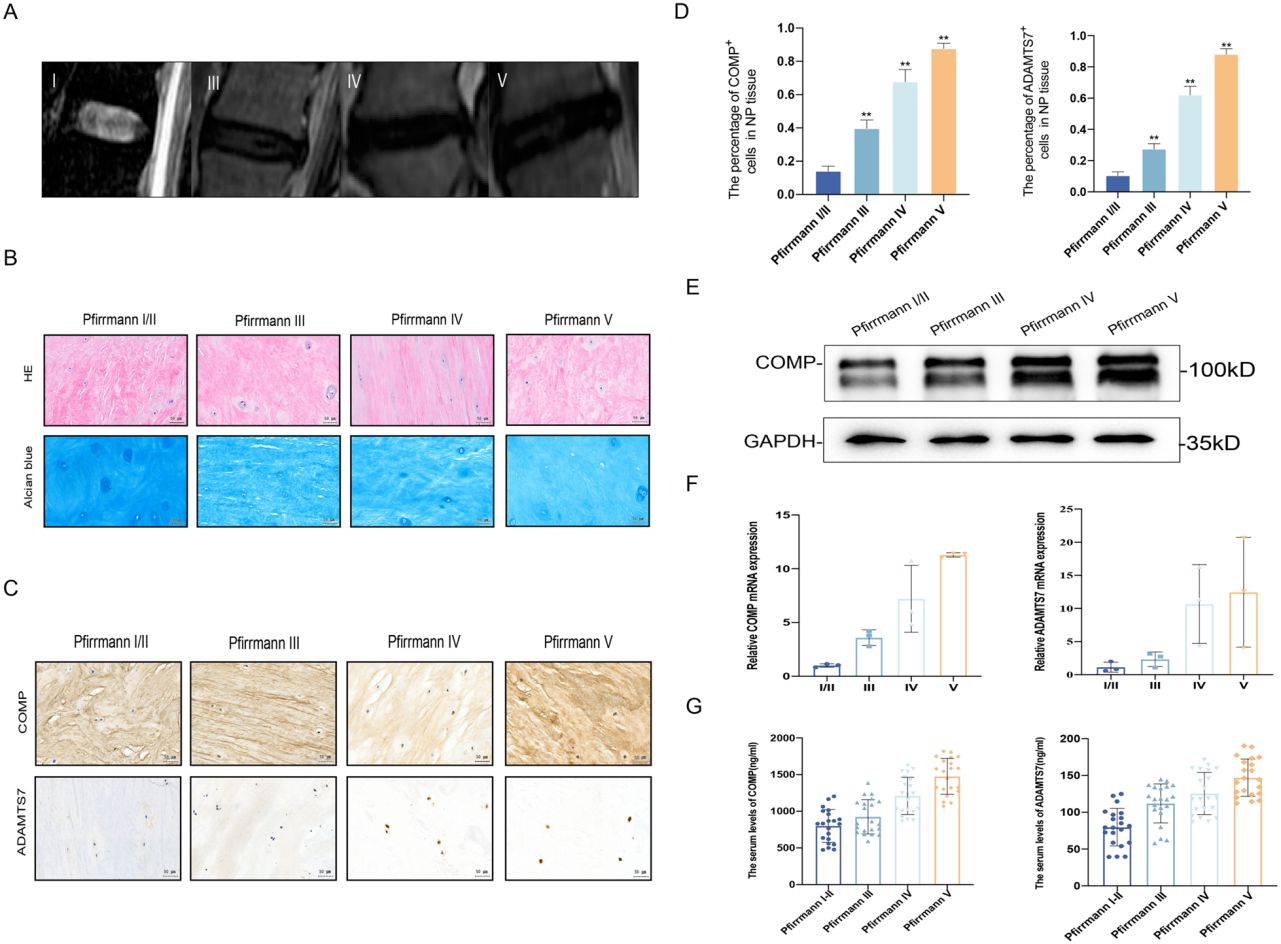
The expression of COMP and ADAMTS7 in patients with different Pfirrmann grades. **A** Pfirrmann grade for IVDD determined using MRI. **B** Representative images of HE staining and Alcian blue staining in NP tissues with different Pfirrmann grades. **C** The expression of COMP and ADAMTS7 in human NP tissues with different Pfirrmann grades determined by IHC. **D** The expression levels of COMP and ADAMTS7 were quantified as positive rate, ^**^ indicating *P* ≤ 0.01 compared to Pfirrmann I/II group. **E** Western blotting analyses of COMP expression in human NP tissues. **F** RT-qPCR analysis of COMP and ADAMTS7 expression in NP tissues at different Pfirrmann grades. **G** The scatterplot of sCOMP concentrations and sADAMTS7 concentrations in patients with different Pfirrmann grades. Scale bar, 50 µm

We recruited a total of LBP patients (*n* = 84 total subjects) including matched group sizes of Pfirrmann grades I/II, III, IV, and V, respectively (*n* = 21 subjects/group; Table 1). Representative classification data are included in Fig. 3A and B, MRI images and histological staining results showing progressive degradation of IVD tissues according to increasing clinical grade. IHC analysis to detect COMP and ADAMTS7 showed progressively upregulation of staining in IVDs with mild (Pfirrmann grade I–II), moderate (Pfirrmann grade III) or severe degeneration (Pfirrmann grade IV–V) (Fig. 3C–D). Validating these findings, the protein levels of COMP detected by Western blotting increased with grade (Fig. 3E), as did the levels of COMP and ADAMTS7 mRNA transcripts measured using qPCR (Fig. 3F). In concert, using ELISA assays we examined the levels of sCOMP and sADAMTS7 in serum samples collected pre-surgically from the cohort. Comparisons among the different Pfirrmann grades showed that both sCOMP and sADAMTS7 concentrations were increased according to IVDD disease severity (Fig. 3G). Thus, the same increased tissue expression and increased circulatory levels of COMP and ADAMTS7 occur during IVDD progression in both the IVDD rabbit model and patients.

**Table 1:** Basic characteristics of population (*n* = 84)

Characteristics	
Age (years)^a^	33.07 ± 14.81
Gender^b^	
Male	47 (56)
Female	37 (44)
BMI (kg/m^2^)^a,b^	23.19 ± 4.22
Normal	46 (54.8)
Overweight/obesity	38 (45.2)
Hypertension^b^	
Yes	12 (14.3)
No	72 (85.7)
Pfirrmann grade^b^	
Levels 1	10(11.9)
Levels 2	11(13.1)
Levels 3	21(25.0)
Levels 4	21(25.0)
Levels 5	21(25.0)
ODI^c^	48 (11–70)
VAS^c^	5 (2–7)
sCOMP (ng/mL)^a^	1102.75 ± 352.44
sADAMTS7 (ng/mL)^a^	116.12 ± 35.75

*BMI* body mass index, *ODI* Oswestry Disability Index, *VAS* visual analog scale

^a^ Data were presented as mean ± standard deviation for continuous variables

^b^ Number (percentage) for categorical variables

^c^ Data were presented as median (P25–P75) for continuous variables

### Regulation and function of COMP levels in the IVD

**Fig. 4:**
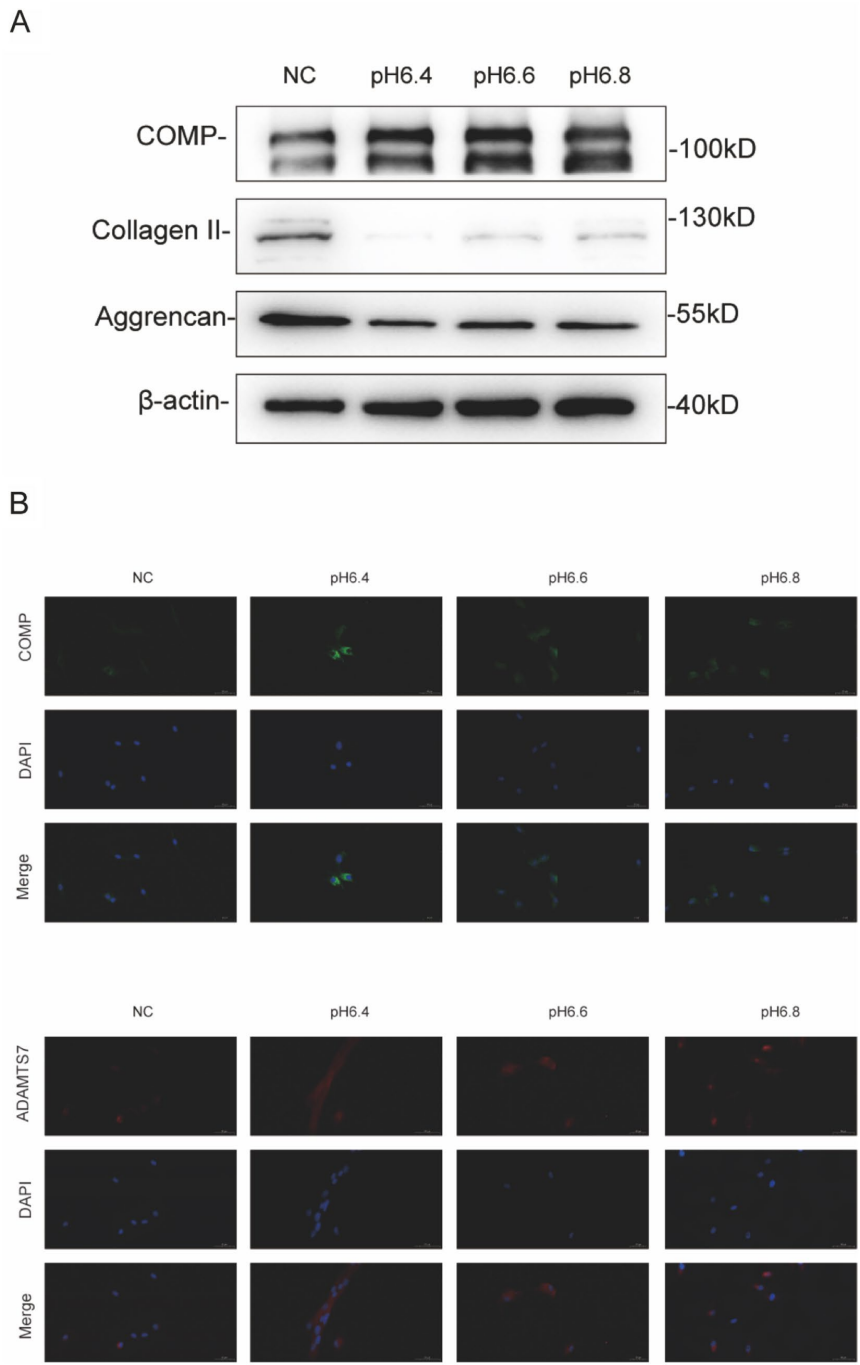
Analyses of COMP and ADAMTS7 expression in NP cells. **A** Western blotting analyses of the expression of COMP, Collagen II and Aggrencan, β-actin serves as loading control. Comparisons between different gradients of extracellular acids group in terms of COMP expression. **B** Immunofluorescence analysis of COMP (Green) and ADAMTS7 (Red) expression under different gradients of extracellular acids

A follow-on question from the preceding data involved how COMP levels were modulated during IVDD pathogenesis. Our previous work focused on the contribution of reduced microenvironmental pH to the degeneration of the IVD [5]. To explore whether this event was implicated in the regulation of COMP and ADAMTS7 levels, we exposed NP cells isolated from patients to acidic conditions to simulate IVD degeneration. Western blotting demonstrated that COMP expression was significantly upregulated in acidified NP cultures while the levels of the ECM components collagen II and aggrecan were reduced (Fig. 4A). Corroborating with these findings, immunofluorescence staining showed increased intensities for COMP as well as ADAMTS7, particularly under pH6.4 conditions (Fig. 4B). These findings implicate microenvironmental acidification as a factor associated with the increased expression of COMP and ADAMTS7 during IVDD pathogenesis. Nevertheless, it was intriguing that the induction of COMP under these conditions appeared unable to counteract mechanisms acting to disrupt ECM components.

**Fig. 5:**
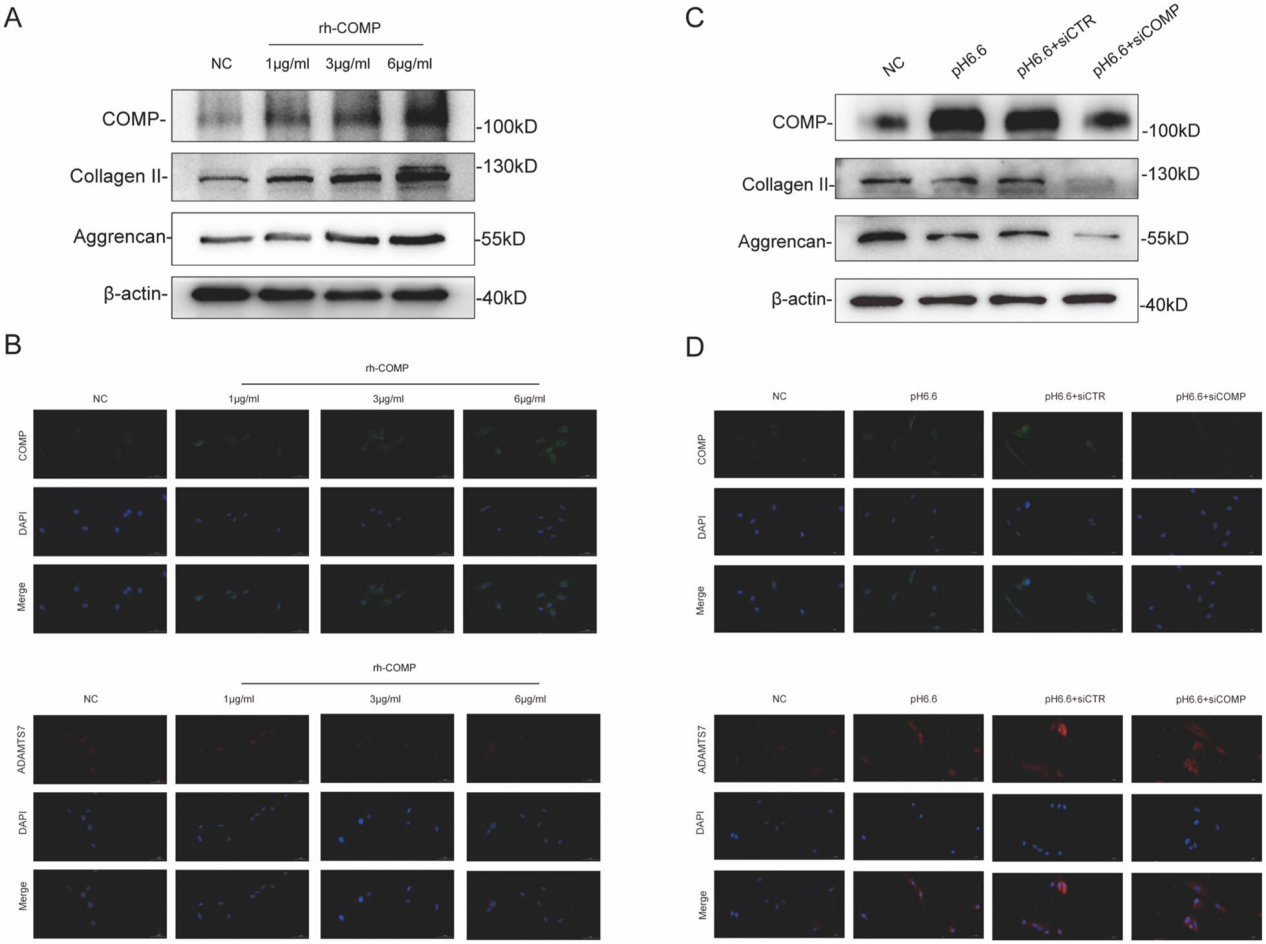
Regulatory role of COMP on ECM degradation and remodeling in NP cells. **A** Western blotting analyses. Comparisons between different concentrations of rhCOMP groups in terms of the expression of COMP and ECM components. **B** The representative images of immunofluorescent staining of COMP (green) and ADAMTS7 (red) in the NP cells after treatment with various concentrations of rhCOMP. **C** Western blotting analysis of the expression of COMP and ECM components in NP cells with or without COMP siRNAs transfection. **D** The representative images of immunofluorescent staining of COMP (green) and ADAMTS7 (red) in the NP cells after with or without si-COMP transfection

To gain further insights into the interplay between COMP, ADAMTS7 and microenvironmental acidification, we experimentally manipulated the levels of COMP. First, we treated NP cells with ectopic recombinant human COMP (rhCOMP) and determined if the levels of ECM components were affected under normal culture conditions. As revealed by Western blotting, rhCOMP exerted a dose-dependent enhancement of collagen II and aggrecan in whole cell lysates (Fig. 5A). The addition of rhCOMP also increased COMP levels in Western blotting and immunofluorescence assays, with increased COMP staining associated with NP cells while the cellular signals for ADAMTS7 were comparatively reduced (Fig. 5B). Subsequently, we subjected NP cells to siRNA-mediated knockdown of COMP or a control siRNA in combination with pH6.6 acidification. As expected, acidification promoted increased COMP levels and reductions in collagen II and aggrecan while depletion COMP exacerbated reductions in their respective levels (Fig. 5C). Immunofluorescence analyses verified the changes in COMP levels while also demonstrating that the increased levels of ADAMTS7 under acidification were also diminished by silencing of COMP (Fig. 5D).

Together these data demonstrate that COMP acts to counteract ECM degradation under steady-state conditions, supporting its essential role in maintaining the integrity of the ECM in the IVD. Associative findings suggest that the actions of COMP appear at least partially linked to ADAMTS7 regulation, but nevertheless, this protective mechanism becomes uncoupled under acidification conditions. Regardless of the underlying phenomena at play, the significant upregulation of COMP and ADAMTS7 in IVDD warranted their further assessment as potential biomarkers.

### Correlative analyses between sCOMP/sADAMTS7 levels and clinical variables in the IVDD patients

Our study of 84 LBP patients included a disproportionate sex representation of males (47 subjects; 56%) to females (37 subjects; 44%). The mean patient age was 33.07 ± 14.81 years with a mean BMI of 23.19 ± 4.22 kg/m^2^ while mean ODI and VAS scores were 48 and 5, respectively. Among the subjects, 12 patients (14.3%) were hypertensive. The mean concentrations of sCOMP and sADAMTS7 in pre-surgical serum samples was 1102.75 ± 352.44 (standard deviation) ng/mL and 116.12 ± 35.75 (standard deviation) ng/mL, respectively (Table 1).

**Fig. 6:**
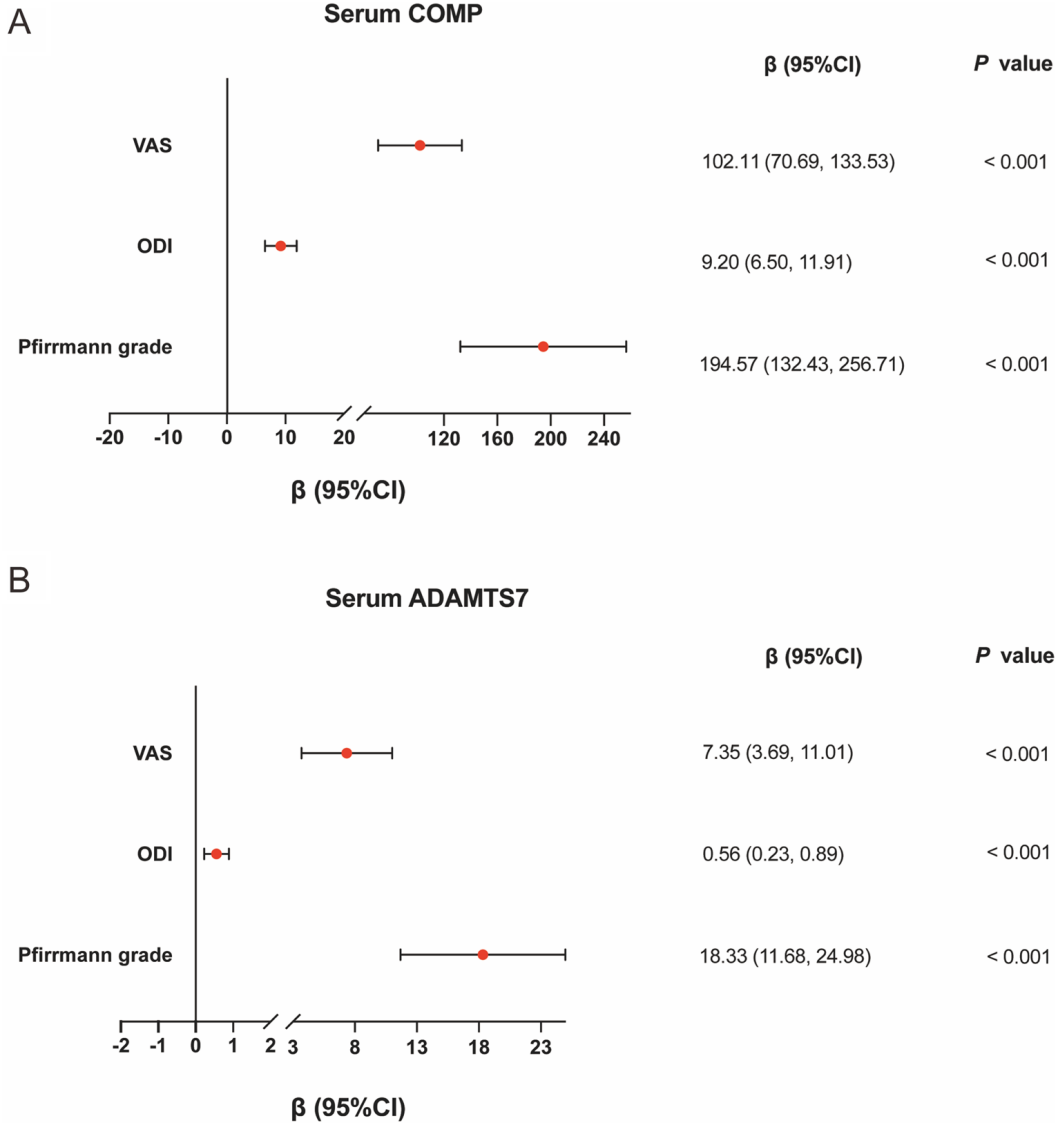
Multivariate linear regressions of VAS score, ODI, Pfirrmann grade and sCOMP concentration (**A**) or sADAMTS7 concentration (**B**). All models were adjusted for age, gender, BMI and hypertension. *VAS* Visual Analog Scale, *ODI* Oswestry Disability Index, *CI* confidence interval

To uncover associations between clinical measures and the levels of sCOMP and sADAMTS7 in IVDD, we applied multivariate linear regressions where the independent variables were assigned as VAS, ODI and Pfirrmann grade, and the outcome variables set as sCOMP and sADAMTS7. After adjustments for the confounding variables of age, gender, BMI and hypertension status, significant positive correlations (*P* < 0.001) were detected between sCOMP levels and VAS score (*β* = 102.11, 95% CI 70.69, 133.53), sCOMP levels and ODI (*β* = 9.20, 95% CI 6.50, 11.90), and sCOMP levels and Pfirrmann Grade (*β* = 194.57, 95% CI 132.43, 256.71) (Fig. 6A). Similarly, VAS score (*β* = 7.35, 95% CI 3.69, 11.01), ODI (*β* = 0.56, 95% CI 0.23, 0.89) and Pfirrmann grade (*β* = 18.33, 95% CI 11.68, 24.98) were found to be positively correlated with sADAMTS7 levels (Fig. 6B).

To analyze whether independent variables (VAS score, ODI and Pfirrmann grade) are also related to the expression of sCOMP or sADAMTS7 in different populations, stratified analyses were used to compare the associations according to gender, BMI, and blood pressure (See Additional file 1: Materials). We found that Pfirrmann grades were both more strongly associated with sCOMP and sADAMTS7 in overweight/obesity group than normal group (*P*-interaction < 0.01), and no other differences were observed in the associations between VAS score, ODI and Pfirrmann grade and sCOMP and sADAMTS7 (all *P* interaction > 0.1).

### Diagnostic potential of sCOMP and sADAMTS7 for IVDD

**Fig. 7:**
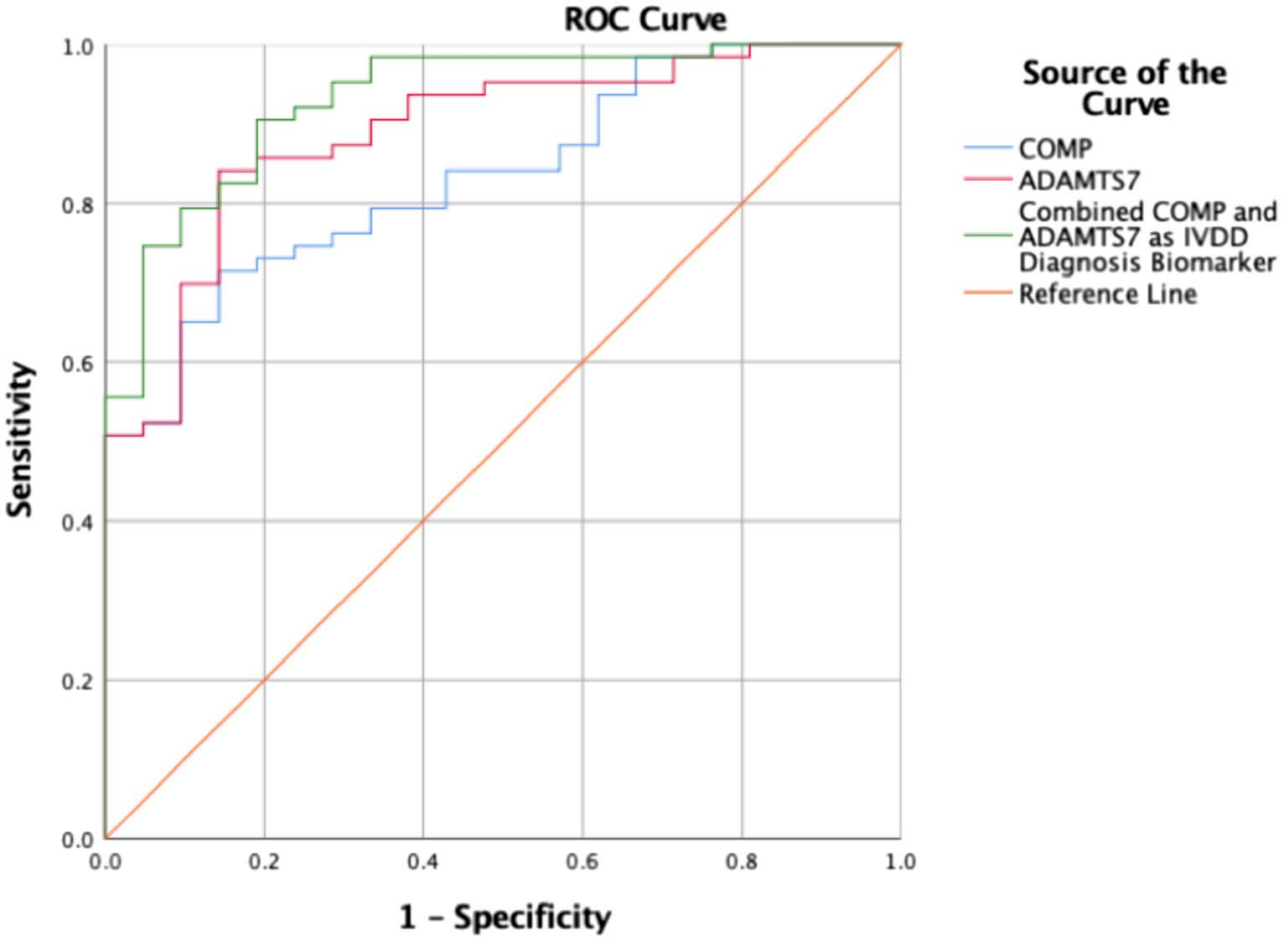
ROC analysis of sCOMP and sADAMTS7. ROC analysis of serum COMP and ADAMTS7 levels discriminating between patients with IVDD (Pfirrmann Grade III–V) and healthy subjects (Pfirrmann Grade I–II)

Given the preceding findings, we implemented ROC analyses to define the diagnostic potential of sCOMP or sADAMTS7 levels in IVDD. The AUC of sCOMP was 0.834 with a 95% CI of 0.747–0.922 while ROC analysis of sCOMP levels to differentiate between healthy subjects (Pfirrmann Grade I–II) and patients with IVDD (Pfirrmann Grade III–V) provided cut-off values of 1021.05 ng/mL (71.4% and 85.7% sensitivity and specificity, respectively). For ADAMTS7, the AUC was 0.889, with a 95% CI of 0.813–0.964. ROC analysis of sADAMTS7 levels provided cut-off values of 102.47 ng/mL (sensitivity 84.1% and specificity 85.7%) to differentiate between healthy subjects (Pfirrmann Grade I–II) and patients with IVDD (Pfirrmann Grade III–V). Notably, the combination of sCOMP and sADAMTS7 test values furthering increasing the AUC to 0.931 with a 95% CI of 0.874–0.989 (Fig. 7 and Table 2). These results indicated that the combinatorial sCOMP and sADAMTS7 tests provide greater authenticity in the diagnosis of IVDD compared to individual measures of sCOMP or sADAMTS7.

**Table 2:** Receiver operating curve analysis of serum COMP and ADAMTS7 levels discriminating between patients with IVDD (Pfirrmann Grade III–V) and healthy subjects (Pfirrmann Grade I-II)

Biomarker(ng/ml)	AUC	Cut-off value	Sensitivity	Specificity	Asymptotic 95% CI	
					Lower bound	Upper bound
COMP	0.834	1021.05	0.714	0.857	0.747	0.922
ADAMTS7	0.889	102.47	0.841	0.857	0.813	0.964
Predicted probability	0.931				0.874	0.989

## Discussion

The diagnosis of IVDD is currently accomplished through radiographic changes and pain caused by herniated discs. Since early interventions are more likely to be effective, the early diagnosis of IVDD is particularly critical. This point has drawn attention to the development of assays for molecular markers derived from cartilage during degradation. In OA and related conditions which are often compared with IVDD, the earliest significant change involves increases in cartilage water content and the increase of permeability to matrix proteoglycans and cartilage degradation products [38]. The latter includes COMP [29, 30] and taking cues from these reports, our study aimed to elaborate on the diagnostic value of COMP as a biochemical marker in IVDD and its association with disease severity and progression.

We first explored the relationship between circulating and tissue COMP levels using a disc degeneration model in rabbits. Here, we observed the remarkable induction of COMP in IVD tissues following damage and the accompanying rise in sCOMP levels. The concentration of sCOMP was significantly elevated 2 weeks after acupuncture and notably, preceded pathological and imaging changes. Although sCOMP levels decreased at 4 weeks, which may be attributed to degradation, it nevertheless remained higher than pre-surgical levels. Similar findings were obtained in Sprague–Dawley rats in a previous study of IVDD [31] but most importantly we were able to substantiate this etiology in patients. In humans with LBP, we found that the concentration of sCOMP was positively correlated with the Pfirrmann classification of IVD, higher sCOMP values with higher pain scores. This is the first report to clearly show that sCOMP is significantly upregulated with increasing clinical and radiological severity in IVDD and moreover, proposes that sCOMP levels reflect the role of COMP in articular cartilage destruction and ECM degradation.

As part of our study, we also conducted a parallel assessment of the role of ADAMTS7. Recently, it has been reported that ADAMTS7 can degrade COMP and they are significantly upregulated in cartilage and synovium tissues from RA patients. Furthermore, the molecular mass of the COMP fragments produced by ADAMTS7 was similar to that of COMP fragments in OA patients [33]. These findings suggest that the COMP degradation observed in OA and RA patients may be related to the upregulation of ADAMTS7. Based on these studies, we found ADAMTS7 upregulation in both IVD tissues and serum accompanied IVDD progression. As such, these observations dovetail with the changes in circulating and tissue COMP levels, suggesting this reflects a common underlying basis during IVDD pathogenesis. In this regard it is noteworthy to consider how COMP and/or ADAMTS7 are able to enter circulation and moreover, why their levels increase during disease progression. Along with expression increases in COMP/ADAMTS7 and rises in cartilage hydration and permeability, there are also early changes in angiogenesis [39, 40]. We speculate that vessel leakiness associated with vascular remodeling cooperate to increase circulating COMP/ADAMTS7 in the early disease stages. In contrast, the rupture of annulus fibrosus during the later stages of disc degeneration [31], providing a patent route for releasing higher levels of COMP/ADAMTS7 into blood. In addition, our results propose further insights into the relationship between COMP and ADAMTS7 during IVDD pathogenesis.

Recent findings show that COMP promotes chondrocyte differentiation and presumably contributes to the repopulation of damaged cartilage areas [41, 42]. This knowledge underscores the importance of understanding how COMP and also ADAMTS7 are regulated during IVDD progression. Their relationship was further evaluated here using in vitro models where the addition of rhCOMP to nucleus pulposus cells stimulated ECM production whereas siRNA knockdown of COMP resulted in the degradative loss of the ECM. These results serve to highlight the protective function COMP but it is nevertheless intriguing that both ADAMTS7 and COMP are upregulated during IVDD progression in vivo. Our in vitro findings suggested that COMP functions to counter increases in ADAMTS7 expression via a transcriptional mechanism. However, this protective function becomes disengaged under acidic culture conditions that mimic the microenvironment of the degenerating IVD. The latter result also provides a possible explanation as to why circulating COMP and ADAMTS7 increase in IVDD.

Last, to evaluate the potential of ADAMTS7 and COMP as IVDD biomarkers, we plotted ROC curves based on serum concentrations of ADAMTS7 and/or COMP. We stratified our cohort into healthy/mild subjects (Pfirrmann Grade I–II) and patent IVDD patients (Pfirrmann Grade III–V) with the resulting area under the curve measurements for sCOMP and sADAMTS7 being 0.834 and 0.889, respectively. Interestingly, these strong characteristics were further improved when sCOMP and sADAMTS7 were analyzed in combination, producing an area under the curve of 0.931, significantly higher than either of the single analyses. Our study also reports cut-off values for sCOMP and sADAMTS7 of 1021.05 and 102.47 ng/ml, respectively, representing the first reported measures for IVDD.

Our study has some limitations which pose as signposts for future investigations. First, a more in-depth understanding of the physical forms and cellular sources of circulating COMP and ADAMTS7 is warranted. This knowledge will help understand, for example, what mechanisms govern the release of COMP and/or ADAMTS7 while also guiding assay development for their respective detection in circulation. The study also needs to be expanded beyond a single center analysis, in particular, subject numbers need to be increased to determine the full discriminatory potential of these assays in reliably detecting early-stage IVD damage. This work will strengthen the case for clinical development of such assays which could also find utility for detecting other types of chondropathies.

## Conclusion

Our overall findings suggest that sCOMP and sADAMTS7 represent potential biomarkers of IVDD, each possessing promising diagnostic characteristics warranting further clinical assessment. On the other hand, the combinatorial findings of our animal and cell-based experiments further advance the understanding of the regulatory relationships that exist between COMP and ADAMTS7.

### Supplementary Information

**Additional file 1:** **Table S1.** The correlation between VAS and sCOMP concentrations in subgroups. **Table S2.** The correlation between ODI and sCOMP concentrations in subgroups. **Table S3.** The correlation between Pfirrmann grade and sCOMP concentrations in subgroups. **Table S4.** The correlation between VAS and sADAMTS7 concentrations in subgroups. **Table S5.** The correlation between ODI and sADAMTS7 concentrations in subgroups. **Table S6.** The correlation between the Pfirrmann grade and sADAMTS7 concentrations in subgroups. **Figure S1.** Study flowchart for the rabbit IVDD model. **Figure S2.** Directed acyclic graph.

## Data Availability

We confirm that all data from this study are available within the article.
