# Supplementary material for: Diagnostic value of serum COMP and ADAMTS7 for intervertebral disc degeneration
Source: Eur J Med Res. 2024 Mar 25;29:196. doi: 10.1186/s40001-024-01784-w (PMC10962093; doi:10.1186/s40001-024-01784-w)
Supplement: Supplementary file 1 — Additional file 1: Table S1. The correlation between VAS and sCOMP concentrations in subgroups. Table S2. The correlation between ODI and sCOMP concentrations in subgroups. Table S3. The correlation between Pfirrmann grade and sCOMP concentrations in subgroups. Table S4. The correlation between VAS and sADAMTS7 concentrations in subgroups. Table S5. The correlation between ODI and sADAMTS7 concentrations in subgroups. Table S6. The correlation between the Pfirrmann grade and sADAMTS7 concentrations in subgroups. Figure S1. Study flowchart for the rabbit IVDD model. Figure S2. Directed acyclic graph. [file 40001_2024_1784_MOESM1_ESM.docx]

**Diagnostic value of Serum COMP and ADAMTS7 for intervertebral disc degeneration**

**Table of Contents**

**Table S1.** The correlation between VAS and sCOMP concentrations in subgroups.

**Table S2.** The correlation between ODI and sCOMP concentrations in subgroups.

**Table S3.** The correlation between Pfirrmann grade and sCOMP concentrations in subgroups.

**Table S4.** The correlation between VAS and sADAMTS7 concentrations in subgroups.

**Table S5.** The correlation between ODI and sADAMTS7 concentrations in subgroups.

**Table S6.** The correlation between the Pfirrmann grade and sADAMTS7 concentrations in subgroups.

**Figure of Contents**

**Figure S1.** Study flowchart for the rabbit IVDD model.

**Figure S2.** Directed acyclic graph.

**Stratified analyses for correlations of VAS score, ODI, Pfirrmann grade and sCOMP and sADAMTS7 concentrations in IVDD**

Regarding sCOMP (ng/mL) (**Table S1-S3**), VAS score, ODI and Pfirrmann grade were positively associated with sCOMP in male (β_VAS_=89.63, 95% CI: 56.33, 122.94; β_ODI_=7.63, 95% CI: 4.69, 10.56; β_Pfirrmann grade_=168.65, 95% CI: 87.18, 250.13), female (β_VAS_=107.96, 95% CI: 52.25, 163.66; β_ODI_=10.81, 95% CI: 6.07, 15.55; β_Pfirrmann grade_=227.50, 95% CI: 112.67, 342.32), BMI normal (β_VAS_=95.71, 95% CI: 63.13, 128.30; β_ODI_=8.29, 95% CI: 5.37, 11.21; β_Pfirrmann grade_=174.30, 95% CI: 91.67, 256.93), overweight/obesity (β_VAS_=106.13, 95% CI: 54.22, 158.04; β_ODI_=9.75, 95% CI: 5.44, 14.07; β_Pfirrmann grade_=247.48, 95% CI: 150.80, 344.16) and normal blood pressure group (β_VAS_=93.61, 95% CI: 64.57, 122.64; β_ODI_=8.62, 95% CI: 6.12, 11.12; β_Pfirrmann grade_=198.94, 95% CI: 126.33, 271.54), but not in hypertension populations (β_VAS_=62.76, 95% CI: -88.51, 214.04; β_ODI_=5.70, 95% CI: -9.00, 20.39; β_Pfirrmann grade_=228.21, 95% CI: -21.10, 477.53), respectively.

For sADAMTS7 (ng/mL), VAS score was positively associated with sADAMTS7 in male (β=4.97, 95% CI: 0.67, 9.28), female (β=7.83, 95% CI: 1.71, 13.96), BMI normal (β=5.08, 95% CI: 0.52, 9.65), overweight/obesity (β=7.54, 95% CI: 1.99, 13.09) and normal blood pressure group (β=5.53, 95% CI: 2.04, 9.02), but not in hypertension group (β=10.70, 95% CI: -4.42, 25.82) (**Table S4**). ODI was positively associated with sADAMTS7 in male (β=0.39, 95% CI: 0.01, 0.77), female (β=0.65, 95% CI: 0.09, 1.21), overweight/obesity (β=0.60, 95% CI: 0.11, 1.09) and normal blood pressure group (β=0.47, 95% CI: 0.16, 0.78), but not in BMI normal(β=0.38, 95% CI: -0.03, 0.79) and hypertension group (β=0.54, 95% CI: -1.12, 2.20) (**Table S5**). Pfirrmann grade was all positively associated with sADAMTS7 in male (β=16.20, 95% CI: 7.26, 25.13), female (β=21.57, 95% CI: 9.79, 33.35), BMI normal (β=13.12, 95% CI: 3.08, 23.17), overweight/obesity (β=26.86, 95% CI: 19.08, 34.65), normal blood pressure (β=17.82, 95% CI: 9.98, 25.66) and hypertension group (β=20.39, 95% CI: 1.63, 39.16) (**Table S6**).

In addition, interaction terms between independent variables (VAS, ODI, Pfirrmann grade) and gender, BMI, hypertension in abovementioned models were also included to examine the potential effect modification by each variable. We found that Pfirrmann grades were both more strongly associated with sCOMP and sADAMTS7 in overweight/obesity group than normal group (*P*-interaction < 0.01), and no other differences were observed in the associations between VAS score, ODI and Pfirrmann grade and sCOMP and sADAMTS7 (all *P*-interaction > 0.1) (**Table S1-S6**).

**Table S1** The correlation between the VAS and sCOMP concentration in subgroups.

| Subgroup | β (95% CI) | *P* value | *P*-interaction |
| --- | --- | --- | --- |
| Gender ^a^ |  |  | 0.927 |
| Male | 89.63 (56.33, 122.94) | < 0.001 |  |
| Female | 107.96 (52.25, 163.66) | < 0.001 |  |
| BMI (kg/m^2^) ^b^ |  |  | 0.273 |
| Normal | 95.71 (63.13, 128.30) | < 0.001 |  |
| Overweight/obesity | 106.13 (54.22, 158.04) | < 0.001 |  |
| Hypertension ^c^ |  |  | 0.618 |
| Normal | 93.61 (64.57, 122.64) | < 0.001 |  |
| Abnormal | 62.76 (-88.51, 214.04) | 0.359 |  |

**Abbreviations:** BMI, body mass index; CI, confidence interval.

^a^ Adjusted for age, BMI and hypertension.

^b^ Adjusted for age, gender and hypertension.

^c^ Adjusted for age, gender, BMI.

**Table S2** The correlation between the ODI and sCOMP concentration in subgroups.

| Subgroup | β (95% CI) | *P* value | *P*-interaction |
| --- | --- | --- | --- |
| Gender ^a^ |  |  | 0.536 |
| Male | 7.63 (4.69, 10.56) | < 0.001 |  |
| Female | 10.81 (6.07, 15.55) | < 0.001 |  |
| BMI (kg/m^2^) ^b^ |  |  | 0.214 |
| Normal | 8.29 (5.37, 11.21) | < 0.001 |  |
| Overweight/obesity | 9.75 (5.44, 14.07) | < 0.001 |  |
| Hypertension ^c^ |  |  | 0.460 |
| Normal | 8.62 (6.12, 11.12) | < 0.001 |  |
| Abnormal | 5.70 (-9.00, 20.39) | 0.390 |  |

**Abbreviations:** BMI, body mass index; CI, confidence interval.

^a^ Adjusted for age, BMI and hypertension.

^b^ Adjusted for age, gender and hypertension.

^c^ Adjusted for age, gender, BMI.

**Table S****3** The correlation between the Pfirrmann grade and sCOMP concentration in subgroups.

| Subgroup | β (95% CI) | *P* value | *P*-interaction |
| --- | --- | --- | --- |
| Gender ^a^ |  |  | 0.963 |
| Male | 168.65 (87.18, 250.13) | < 0.001 |  |
| Female | 227.50 (112.67, 342.32) | < 0.001 |  |
| BMI (kg/m^2^) ^b^ |  |  | 0.003 |
| Normal | 174.30 (91.67, 256.93) | < 0.001 |  |
| Overweight/obesity | 247.48 (150.80, 344.16) | < 0.001 |  |
| Hypertension ^c^ |  |  | 0.395 |
| Normal | 198.94 (126.33, 271.54) | < 0.001 |  |
| Abnormal | 228.21 (-21.10, 477.53) | 0.065 |  |

**Abbreviations:** BMI, body mass index; CI, confidence interval.

^a^ Adjusted for age, BMI and hypertension.

^b^ Adjusted for age, gender and hypertension.

^c^ Adjusted for age, gender, BMI.

**Table S4** The correlation between the VAS and sADAMTS7 concentration in subgroups.

| Subgroup | β (95% CI) | *P* value | *P*-interaction |
| --- | --- | --- | --- |
| Gender ^a^ |  |  | 0.667 |
| Male | 4.97 (0.67, 9.28) | 0.025 |  |
| Female | 7.83 (1.71, 13.96) | 0.014 |  |
| BMI (kg/m^2^) ^b^ |  |  | 0.214 |
| Normal | 5.08 (0.52, 9.65) | 0.030 |  |
| Overweight/obesity | 7.54 (1.99, 13.09) | < 0.01 |  |
| Hypertension ^c^ |  |  | 0.785 |
| Normal | 5.53 (2.04, 9.02) | < 0.01 |  |
| Abnormal | 10.70 (- 4.42, 25.82) | 0.138 |  |

**Abbreviations:** BMI, body mass index; CI, confidence interval.

^a^ Adjusted for age, BMI and hypertension.

^b^ Adjusted for age, gender and hypertension.

^c^ Adjusted for age, gender, BMI.

**Table S5** The correlation between the ODI and sADAMTS7 concentration in subgroups.

| Subgroup | β (95% CI) | *P* value | *P*-interaction |
| --- | --- | --- | --- |
| Gender ^a^ |  |  | 0.607 |
| Male | 0.39 (0.01, 0.77) | 0.045 |  |
| Female | 0.65 (0.09, 1.21) | 0.025 |  |
| BMI (kg/m^2^) ^b^ |  |  | 0.263 |
| Normal | 0.38 (-0.03, 0.79) | 0.069 |  |
| Overweight/obesity | 0.60 (0.11, 1.09) | 0.018 |  |
| Hypertension ^c^ |  |  | 0.354 |
| Normal | 0.47 (0.16, 0.78) | < 0.01 |  |
| Abnormal | 0.54 (-1.12, 2.20) | 0.465 |  |

**Abbreviations:** BMI, body mass index; CI, confidence interval.

^a^ Adjusted for age, BMI and hypertension.

^b^ Adjusted for age, gender and hypertension.

^c^ Adjusted for age, gender, BMI.

**Table S6** The correlation between the Pfirrmann grade and sADAMTS7 concentration in subgroups.

| Subgroup | β (95% CI) | *P* value | *P*-interaction |
| --- | --- | --- | --- |
| Gender ^a^ |  |  | 0.674 |
| Male | 16.20 (7.26, 25.13) | < 0.01 |  |
| Female | 21.57 (9.79, 33.35) | < 0.01 |  |
| BMI (kg/m^2^) ^b^ |  |  | < 0.001 |
| Normal | 13.12 (3.08, 23.17) | 0.012 |  |
| Overweight/obesity | 26.86 (19.08, 34.65) | < 0.001 |  |
| Hypertension ^c^ |  |  | 0.715 |
| Normal | 17.82 (9.98, 25.66) | < 0.001 |  |
| Abnormal | 20.39 (1.63, 39.16) | 0.038 |  |

**Abbreviations:** BMI, body mass index; CI, confidence interval.

^a^ Adjusted for age, BMI and hypertension.

^b^ Adjusted for age, gender and hypertension.

^c^ Adjusted for age, gender, BMI.


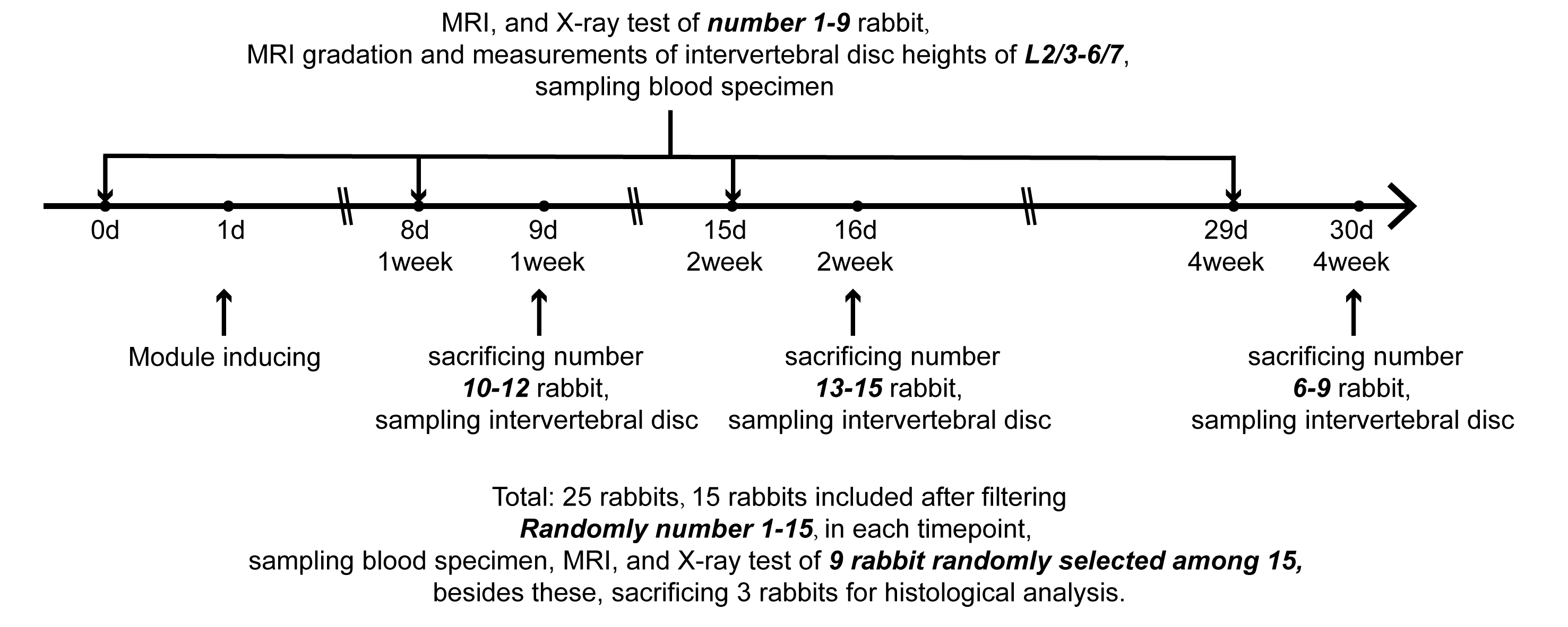


**Figure S1.** Study flowchart for the rabbit IVDD model.


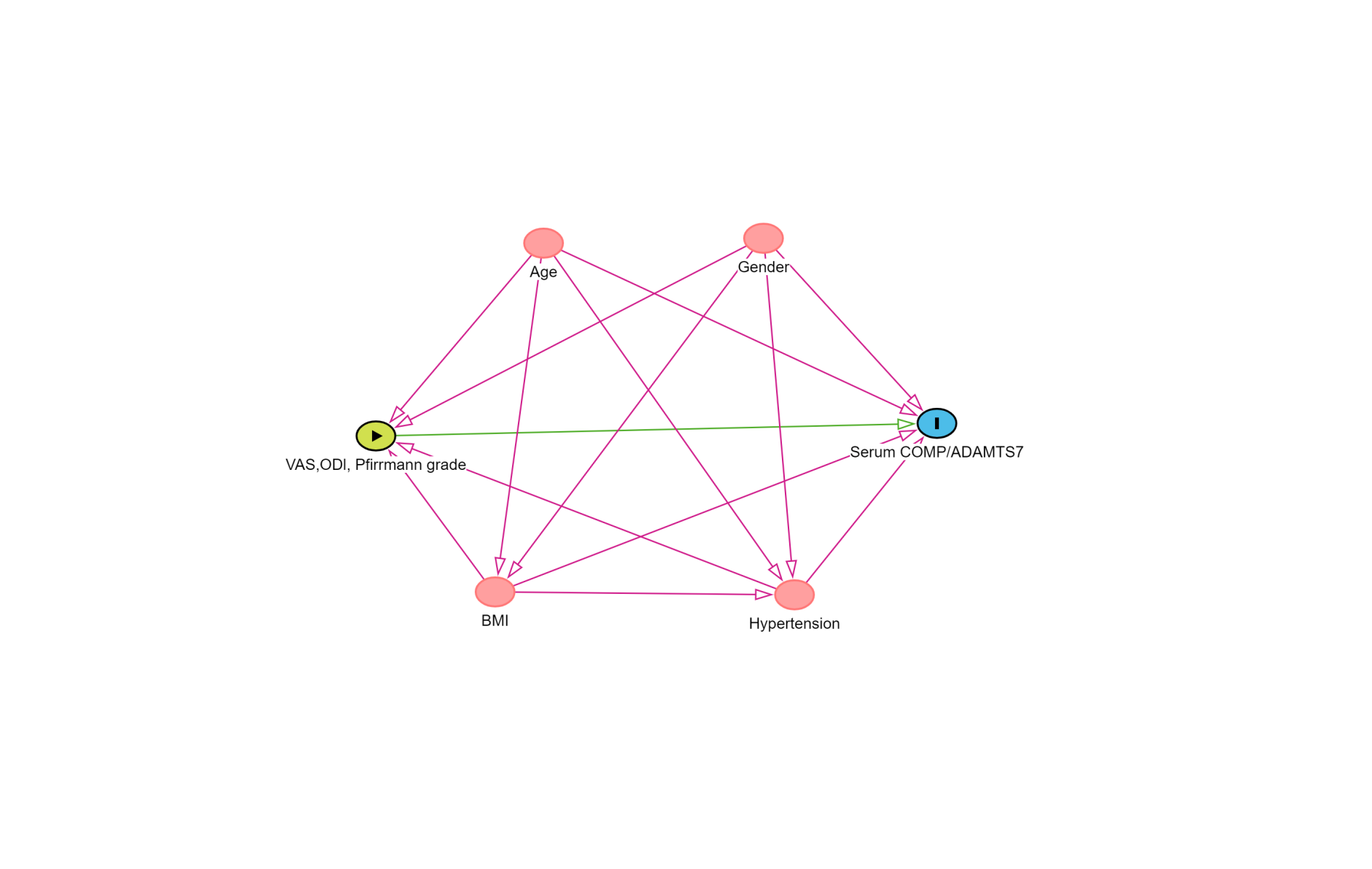


**Figure S2.** Directed acyclic graph of association between serum COMP/ADAMTS7 and VAS, ODI, Pfirrmann grade.
